# Aneuploidy enables cross-tolerance to unrelated antifungal drugs in *Candida parapsilosis*

**DOI:** 10.3389/fmicb.2023.1137083

**Published:** 2023-04-11

**Authors:** Liu-liu Sun, Hao Li, Tian-hua Yan, Yong-bing Cao, Yuan-ying Jiang, Feng Yang

**Affiliations:** ^1^Department of Pharmacy, Shanghai Tenth People's Hospital, Tongji University School of Medicine, Shanghai, China; ^2^Department of Physiology and Pharmacology, School of Basic Medicine and Clinical Pharmacy, China Pharmaceutical University, Nanjing, China; ^3^Department of Vascular Diseases, Shanghai TCM-Integrated Hospital, Shanghai University of Traditional Chinese Medicine, Shanghai, China

**Keywords:** *Candida parapsilosis*, caspofungin, aneuploidy, cross tolerance, 5-flucytosine

## Abstract

*Candida parapsilosis* is an emerging major human fungal pathogen. Echinocandins are first-line antifungal drugs for the treatment of invasive *Candida* infections. In clinical isolates, tolerance to echinocandins in *Candida* species is mostly due to point mutations of *FKS* genes, which encode the target protein of echinocandins. However, here, we found chromosome 5 trisomy was the major mechanism of adaptation to the echinocandin drug caspofungin, and *FKS* mutations were rare events. Chromosome 5 trisomy conferred tolerance to echinocandin drugs caspofungin and micafungin and cross-tolerance to 5-flucytosine, another class of antifungal drugs. The inherent instability of aneuploidy caused unstable drug tolerance. Tolerance to echinocandins might be due to increased copy number and expression of *CHS7*, which encodes chitin synthase. Although copy number of chitinase genes *CHT3* and *CHT4* was also increased to the trisomic level, the expression was buffered to the disomic level. Tolerance to 5-flucytosine might be due to the decreased expression of *FUR1*. Therefore, the pleiotropic effect of aneuploidy on antifungal tolerance was due to the simultaneous regulation of genes on the aneuploid chromosome and genes on euploid chromosomes. In summary, aneuploidy provides a rapid and reversible mechanism of drug tolerance and cross-tolerance in *C. parapsilosis*.

## Introduction

In recent years, due to HIV infection, solid organ and stem cell transplantation, intensified chemotherapy, and immunosuppression for autoimmune diseases, the at-risk immunocompromised population is steadily increasing. Opportunistic fungal infections are emerging as an important public health concern and cost burden (Benedict et al., 2019). *Candida, Aspergillus*, and *Mucor* species are the most frequently isolated fungi in immunocompromised patients (Badiee and Hashemizadeh, 2014). *Candida* infections are most often caused by *C. albicans* (Pappas et al., 2018), but non-albicans *Candida* (NAC) species, such as *C. glabrata, C. parapsilosis, C. tropicalis*, and *C. krusei*, are also increasingly reported as a cause of superficial infections as well as disseminated and deep-tissue infections (Whaley et al., 2016). However, currently, there are only four classes of antifungal drugs: polyenes, azoles, 5-flucytosine, and echinocandins. Echinocandins are the first-line drugs for the treatment of bloodstream *Candida* infections (Pappas et al., 2016).

In yeasts, the target protein of echinocandins is encoded by *FKS* genes. Mutations of *FKS* genes, especially in the hot spot regions, are the major cause of resistance to echinocandins [reviewed in Perlin (2015)]. In *C. parapsilosis*, caspofungin (CSP)-resistant clinical isolates (Chen et al., 2010; Pfeiffer et al., 2010; Pfaller et al., 2011; Siopi et al., 2022) and mutants obtained during *in vitro* evolution (Chassot et al., 2016; Papp et al., 2018; Arastehfar et al., 2021) also usually bear mutations in hot spot regions of *FKS* genes. But in some isolates, resistance is due to mutations outside of the hot spot regions or there are no mutations in *FKS* genes (Berrio et al., 2018). Furthermore, mutations outside of the hot spot regions of *FKS* genes have been associated with increased tolerance, but not resistance to echinocandins (Daneshnia et al., 2022).

5-flucytosine (5FC) is a prodrug. It enters fungal cells *via* the cytosine permease. Then, it is converted into toxic 5-fluorouracil (5FU) by cytosine deaminase. 5FU is further processed by uracil phosphoribosyltransferase, and the product inhibits both DNA and protein synthesis (Vermes et al., 2000). In the *C. parapsilosis* genome, cytosine permease, cytosine deaminase, and uracil phosphoribosyltransferase are encoded by *FCY2/CPAR2_806580, FCA1*/*CPAR2_602820*, and *FUR1*/*CPAR2_502030*, respectively. 5FC is active against *Candida* species and *Cryptococcus* species, but monotherapy of 5FC usually results in the rapid development of resistance, and the resistance is usually due to loss-of-function mutations of genes involved in the uptake and intracellular metabolism of 5FC (Whelan, 1987; Hope et al., 2004; Papon et al., 2007; Billmyre et al., 2020; Chang et al., 2021). *C. parapsilosis* clinical isolates are generally susceptible to 5FC (Barchiesi et al., 2000; Cuenca-Estrella et al., 2001). The report of 5FC resistance in *C. parapsilosis* is very limited, possibly because the monotherapy of 5FC against *C. parapsilosis* infections is not recommended in the clinic.

In addition to genetic mutations, aneuploidy, which is a cellular state of unbalanced chromosome copy number, is considered a prevalent strategy of rapid adaptation to stresses including antifungal agents in fungal pathogens [reviewed in Tsai and Nelliat (2019)]. For example, in *C. albicans*, different stresses can select the same aneuploidy, thereby causing cross-adaptation (Yang et al., 2013, 2017, 2019, 2021b). Furthermore, in addition to the direct regulation of genes on the aneuploid chromosome (Kabir et al., 2005; Suwunnakorn et al., 2016; Yang et al., 2019, 2021b), aneuploidy also indirectly regulates genes on euploid chromosomes. For example, the copy number of Chr5 negatively regulates the expression of *SOU1* on Chr4. *SOU1* encodes a sorbose reductase required for L-sorbose utilization (Greenberg et al., 2005). Therefore, in the Chr5x1 strain, *SOU1* is upregulated, thereby enabling *C. albicans* to utilize L-sorbose as the sole carbon source (Janbon et al., 1998). However, in *C. parapsilosis*, reports of aneuploidy formation are very limited. Previously we found aneuploidy was the predominant mechanism of adaptation to ER stress inducer tunicamycin and sphingolipid biosynthesis inhibitor aureobasidin A in *C. parapsilosis*. Furthermore, both stresses selected Chr6x3 adaptors, and Chr6x3 conferred cross-tolerance to tunicamycin and aureobasidin A (Yang et al., 2021c). The role of aneuploidy in the adaptation of *C. parapsilosis* to commonly used antifungal drugs is still largely unknown.

Antifungal resistance is usually defined as “the ability to grow at antifungal drug concentrations above a defined antifungal susceptibility breakpoint”. The extent of resistance can be measured by broth microdilution assay, which determines the minimal inhibitory concentration (MIC) of antifungals that inhibits fungal growth (Fisher et al., 2022). Disk diffusion assay (DDA) is an official method and is one of the most widely used methods in many clinical microbiology laboratories for routine antimicrobial sensitivity testing. According to the standards published by the Clinical and Laboratory Standards Institute (CLSI), in a DDA experiment, agar plates are inoculated with a standardized number of cells. A paper disk containing the test chemical is placed on the surface of the medium. After the incubation of the plates under suitable conditions, the diameters of the zone of inhibition (ZOI) are measured (CLSI, 2012). This method is fast, simple, and low cost, and the results are easy to interpret. However, growth inside the ZOI is not considered.

Recently, Judith Berman lab developed a new method to analyze and interpret the DDA results. They performed DDAs using disks containing fluconazole, a fungistatic drug against *C. albicans*. They defined the growth of subpopulations of *C. albicans* cells inside the ZOI as “antifungal tolerance” (Rosenberg et al., 2018). By definition, antifungal tolerance is “a characteristic of drug-susceptible genotypes to grow slowly at or above inhibitory drug concentrations” (Berman and Krysan, 2020; Fisher et al., 2022). A pipeline called *diskImageR* was developed to analyze DDA data (Gerstein et al., 2016; Rosenberg et al., 2018; Xu et al., 2021). *diskImageR* quantifies the radius (RAD) of the ZOI as a parameter that relates to the MIC, and the fraction of growth (FoG) within the ZOI as a parameter that measures tolerance. RAD_20_, the RAD value corresponding to the point where 20% growth reduction occurs, and FoG_20_, the area under the curve at the RAD threshold, divided by the maximum growth, were usually used as measurements of resistance and tolerance, respectively (Gerstein et al., 2016; Rosenberg et al., 2018; Xu et al., 2021). However, for fungicidal drugs, such as CSP, growth in the presence of the drug without change in RAD or FoG is also considered tolerance (Berman and Krysan, 2020).

In this study, we investigated how *C. parapsilosis* adapted to CSP and the impact of adaptation on tolerance to other antifungal drugs. We randomly analyzed 30 adaptors and we got mainly chromosome 5 trisomy (Chr5x3, *n* = 29) and occasionally chromosome 1 trisomy (Chr1x3, *n* = 1). Genetic mutation of *FKS* genes was not detected. Chr5x3 conferred tolerance, not resistance, to echinocandin drugs CSP and micafungin (MCF), as well as cross-tolerance to 5FC, but it also caused hypersensitivity to fluconazole (FLC). The Chr5x3 adaptor was unstable. It spontaneously reverted to chromosome 5 disomy (Chr5x2), and tolerance to echinocandins and 5FC was concomitantly lost. Aneuploidy simultaneously upregulated the expression of genes on the aneuploid chromosome and genes on other chromosomes, including genes associated with tolerance to echinocandins, 5FC and FLC. Further exposure of one Chr1x3 adaptor to CSP also selected mostly Chr5x3 adaptors, thereby causing cross-tolerance to echinocandins and 5FC. Therefore, we posit that Chr5x3 provides a rapid and reversible strategy of adaptation to CSP and cross-adaptation to 5FC in *C. parapsilosis*.

## Materials and methods

### Strains and growth conditions

The strains used in this study are listed in Supplementary Table 1. *C. parapsilosis* clinical isolate #12108 was used as the wild-type strain. The stock culture was preserved in 25% of glycerol and maintained at −80°C. Cells were routinely grown in the yeast extract–peptone–dextrose (YPD) media (1% [w/v] yeast extract, 2% [w/v] peptone, and 2% [w/v] D-glucose) at 37°C in a shaking incubator at 150–200 rpm. For solid medium, 2% [w/v] agar was added. SD agar plates (0.67% [wt/vol] yeast nitrogen base without amino acids, 2% [wt/vol] D-glucose, and 2% [wt/vol] agar) were used for testing tolerance to 5FC. Drugs were dissolved in dimethyl sulfoxide (DMSO) and stored at −20°C. The concentrations of echinocandins, 5FC, and FLC were 10, 0.5, and 40 mg/ml, respectively.

### Obtaining caspofungin adaptors

Cells were suspended in distilled water. Cell density was determined by using a hemocytometer and was adjusted to 1 × 10^7^ cells/ml. A total of 100 μl of cell suspension were spread on YPD plates supplemented with 100, 200, and 400 ng/ml of CSP. On day 5, only ~368 colonies (adaptors) appeared on the plate with 400 ng/ml of CSP, while on other plates, we saw lawn growth. A total of 30 adaptors (TJ60–TJ89) were randomly chosen from the plate with 400 ng/ml of CSP. Each adaptor was streaked from the drug plate onto the YPD plate. The plates were incubated at 37°C for 3 days. Four to six colonies with similar sizes were selected and frozen in 1 ml of 25% glycerol at −80°C.

### Spot assay

Cells were suspended in distilled water and adjusted to 1 × 10^7^ cells/ml. A total of 3 μl of 10-fold serial dilutions were spotted on YPD or SD plates with or without drugs (control) at 37°C and photographed after 3 days.

### Growth curves

Cells were suspended in YPD broth. Cell densities were adjusted to 2.5 × 10^3^ cells/ml in YPD broth with or without test drugs in a 96-well plate. The plate was incubated at 37°C. OD_595_ was monitored in a Tecan plate reader (Infinite F200 PRO, Tecan, Switzerland) at 15 min time intervals for 48 h. Data are represented as the mean ± SD of three biological replicates.

### Colony instability assay

As described previously (Yang et al., 2021d), Chr1x3 and Chr5x3 adaptors were streaked from−80°C freezer to YPD agar and incubated at 37°C for 72 h. One small colony was randomly chosen and suspended in distilled water. Cells were diluted with distilled water and ~200 cells were spread on a YPD plate and incubated at 37°C for 72 h. One small (S) colony and one large (L) colony were randomly chosen for further studies.

### Disk diffusion assays

The CLSI M44-A2 guidelines (CLSI, 2012) for the antifungal disk diffusion susceptibility testing were followed with slight modifications. Strains were grown on agar plates, and cell density was adjusted to 1 × 10^6^ cells/ml as described earlier. A total of 100 μl of cell suspension was plated on plates. One paper disk (GE Healthcare, USA) was placed in the center of each plate. The plates were then incubated for 72 h and photographed.

### DNA-seq

The test strains were grown on YPD plates at 37°C at a density of ~200 colonies per plate. Colonies were collected by centrifugation in a microfuge at 3,000 rpm for 1 min. Genomic DNA was extracted manually using the phenol–chloroform method (Selmecki et al., 2015). The genomic DNA library was prepared by BGI (Wuhan, China) according to their standard preparation protocol. Approximately 1 μg of genomic DNA was randomly fragmented with a Covaris LE220. Fragments (300–400 bp) were selected, end-repaired, and 3' adenylated using the Agencourt AMPure XP-Medium kit, then ligated to adaptors. The ligation products were amplified by PCR. After purification, the PCR products were heat denatured, then circularized with a splint oligo sequence. The single-strand circular DNA (ssCirDNA) was formatted as the final library, qualified by QC, and then sequenced by BGISEQ-500. ssCir DNA molecules formed a DNA nanoball (DNB) containing more than 300 copies through rolling-cycle replication. The DNBs were loaded into a patterned nano array by using high-density DNA nanochip technology and were sequenced on the BGISEQ-500 platform using BGISEQ-500 high-throughput sequencing kit (PE100). Finally, pair-end 100 bp reads were obtained by combinational probe-anchor synthesis (cPAS). Raw FASTQ files were uploaded to YMAP (version 1.0) (http://lovelace.cs.umn.edu/Ymap/) (Abbey et al., 2014). Read depth was plotted as a function of chromosome position using the CDC317 reference genome of *C. parapsilosis* (http://www.candidagenome.org/download/sequence/C_parapsilosis_CDC317/current/). Contig005504, contig005569, contig005806, contig005807, contig005809, contig006110, contig006139, and contig006372 were renamed to chromosome 1, chromosome 2, chromosome 3, chromosome 4, chromosome 5, chromosome 6, chromosome 7, and chromosome 8, respectively. Chromosome end bias and GC content bias were corrected by YMAP (Abbey et al., 2014).

### RNA-seq

RNA-seq was performed as described previously (Yang et al., 2021b). Strains were streaked onto YPD plates from the −80°C freezer. After 72 h of incubation at 37°C, several colonies of similar sizes were chosen. Colonies were suspended in distilled water and adjusted to 1 × 10^4^ cells/ml. A total of 100 μl of cell suspension were spread on YPD plates. The plates were incubated at 37°C for 72 h. Cells were collected by centrifugation, washed, and flash-frozen in liquid nitrogen. The total RNA was extracted for nine independent samples, corresponding to three conditions and three biological replicates. Total RNA extraction and purification, library construction, and sequencing were performed as described in Yang et al. (2013). Raw sequence files (.fastq files) underwent quality control analysis using the FastQC tool (http://www.bioinformatics.babraham.ac.uk/projects/fastqc). Reads were mapped to the *C. parapsilosis* CDC317 reference genome (http://www.candidagenome.org/download/sequence/C_parapsilosis_CDC317/current/). Differential gene expression profiling was carried out using DESeq2 (Love et al., 2014) with standard parameters. Genes with false discovery rate (FDR)-adjusted *P*-value (<0.05) and expression fold changes of more than 1.3 or <-1.3 were considered differentially expressed.

### Reverse transcriptase PCR (RT-PCR)

Cells were grown under the same experimental conditions as in RNA-seq. RT-qPCR was performed in 96-well plates (Bio-Rad) on the CFX Touch 96-well Real-Time Systems (Bio-Rad). Primer sequences are listed in Supplementary Table 2. The reaction mix was performed using 5 μl of iTaq Universal SYBR Green Supermix (Bio-Rad), 2 μl of 2 μM primer mix, 2 μl of a diluted 1:10 cDNA, and water to make up the final volume to 10 μl. Cycling conditions were 95 °C for 3 min, 40 cycles of 95 °C for 5 s, and 60 °C for 30 min. Melt curve analysis conditions were 5 s at 95°C and then 5 s each at 0.5°C increments between 60°C and 95°C. *ACT1* (*CPAR2_201570*) was the internal control. Fold change was calculated using the 2^−ΔΔCt^ method (Livak and Schmittgen, 2001). All RT-PCR experiments were performed using three biological and three technical replicates.

### Statistical analysis

The significance of differences between growth curves was performed using Tukey's honest significant difference (HSD) test.

## Results

### Aneuploidy enables adaptation to the lethal amount of caspofungin in *C. parapsilosis*

**Figure 1:**
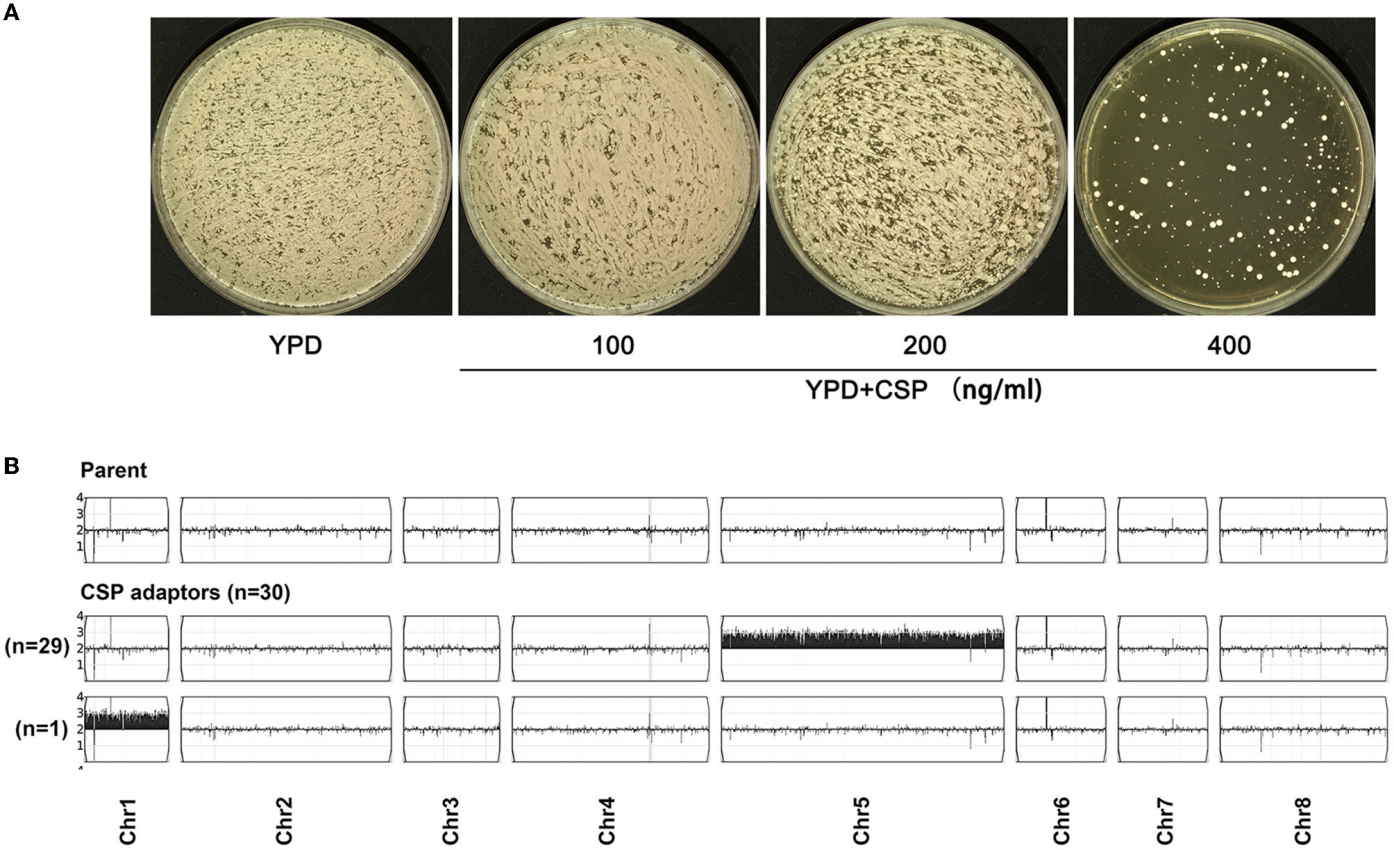
In *C. parapsilosis*, caspofungin adaptors are aneuploid. Approximately 1 million cells of *C. parapsilosis* strain #12108 were spread on YPD plates with or without caspofungin (CSP). The plates were incubated at 37°C for 5 days **(A)**. On the plate with 400 ng/ml of CSP, randomly 30 adaptors were chosen and sequenced. The karyotypes were visualized using Ymap **(B)**. Read depth (normalized to that of the diploid parent) is shown on the y-axis on a log_2_ scale converted to absolute copy numbers (1–4). The number of adaptors bearing the same aneuploidy was also shown in the figure.

In order to obtain CSP adaptors, ~1 million cells of *C. parapsilosis* clinical isolate #12108 were spread on the YPD plate supplemented with 400 ng/ml of CSP. After 5 days of incubation at 37°C, ~368 colonies (adaptors) were obviously visible on the plate (Figure 1A). We randomly picked up 30 adaptors (TJ60–TJ89, Supplementary Figure 1) with different colony sizes on the drug plate. Whole genome sequencing indicated that all the adaptors were aneuploid: 29 had trisomy of chromosome 5 (Chr5x3), and 1 (TJ74) had trisomy of chromosome 1 (Chr1x3) (Figure 1B). Of note, there are three *FKS* genes in *C. parapsilosis* genome: *GSC1*/*CPAR2_106400, GSL1*/*CPAR2_109680*, and *GSL2*/*CPAR2_804030*. Sequences of these three *FKS* genes were visualized in Integrative Genomics Viewer (IGV), and we did not detect any mutation in the 30 adaptors. *GSC1* and *GSL1* are on Chr2, and *GSL2* is on Chr4, but none of the adaptors had aneuploidy of Chr2 or Chr4. Therefore, Chr5x3 was the major mechanism of adaptation to CSP. Mutations of *FKS* genes and aneuploidy of chromosomes on which the *FKS* genes reside were not detected.

### Fitness loss and gain in caspofungin adaptors

**Figure 2:**
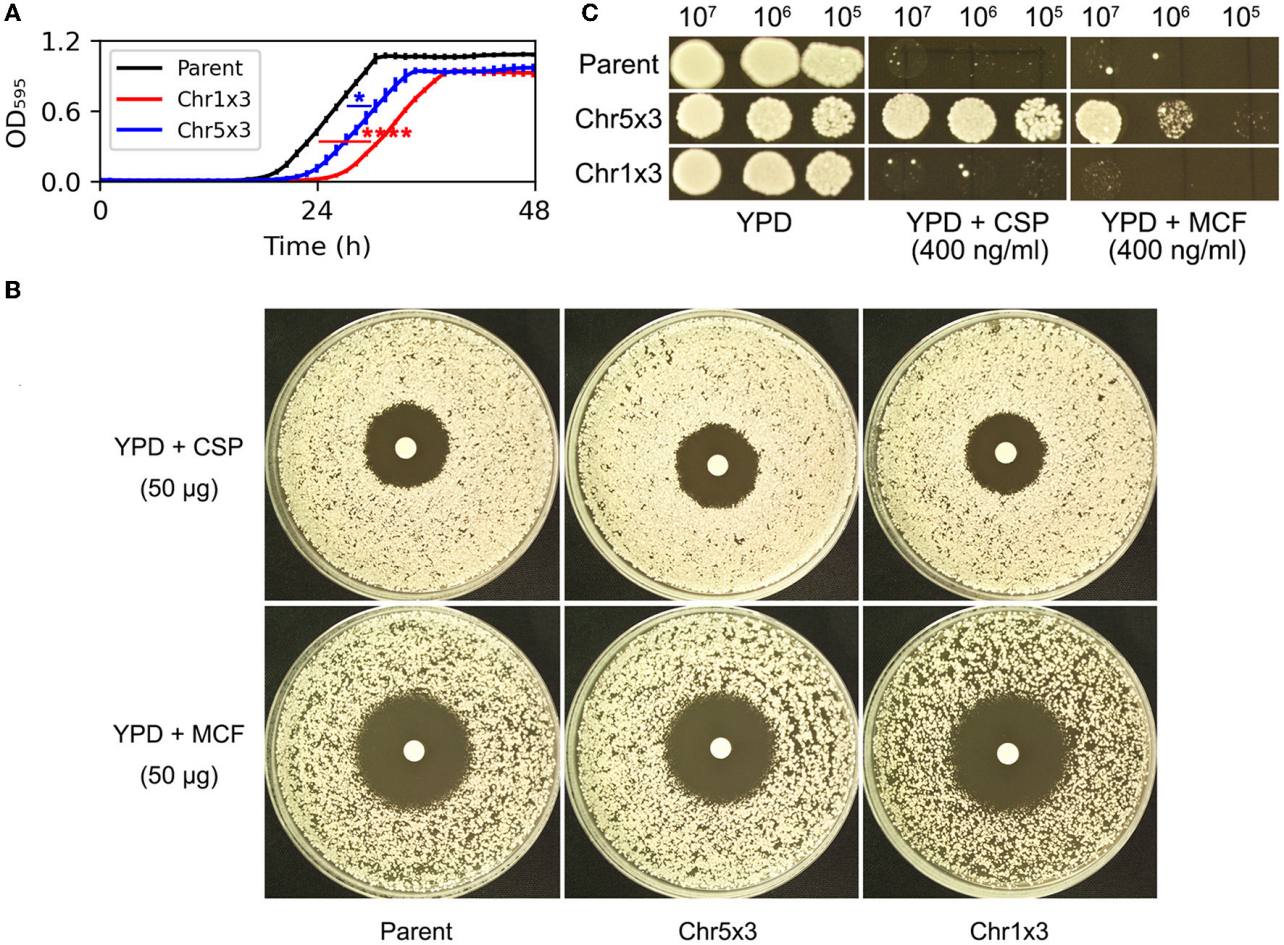
Fitness loss and gain in caspofungin adaptors. The fitness cost of adaptation to caspofungin (CSP) was evaluated by growing strains in YPD broth without stress **(A)**. Optical density at 595 nm (OD_595_) was measured every 1 h for 48 h at 37°C using a Tecan plate reader (Infinite F200 PRO, Tecan, Switzerland). Data are represented as the mean ± SD of three biological repeats. * and **** indicate *p*-values <0.05, and *p* < 0.0001, respectively, as determined by Tukey's HSD test. In **(B)**, disk diffusion assays were performed to compare Chr1x3 and Chr5x3 adaptors to parent for resistance to CSP and micafungin (MCF). The paper disks contained 50 μg of CSP or MCF as indicated in the figure. In **(C)**, 3 μl of 10-fold serial dilutions were spotted on YPD plates. Drug concentrations are shown in the figure. In **(B)** and **(C)**, the plates were incubated at 37°C for 72 h and then photographed.

It is known that in *Saccharomyces cerevisiae* and *C. albicans*, aneuploids usually have fitness loss in rich medium in the absence of stress (Pavelka et al., 2010; Yang et al., 2021d). In *C. albicans*, gain-of-function mutations of genes associated with resistance to azoles also have fitness costs *in vitro* and *in vivo* (Sasse et al., 2012; Hill et al., 2015; Popp et al., 2017). We asked if the aneuploid CSP adaptors also had fitness loss. Growth curves of the adaptors and the parent were measured in YPD broth (Figure 2A). The Chr5x3 adaptor TJ60 and the Chr1x3 adaptor TJ74 were significantly less fit than the parent (*p* < 0.0001 and *p* < 0.05, respectively. Tukey's HSD test).

We investigated whether the adaptors gained resistance or tolerance to CSP. DDAs with disks containing CSP were performed. In DDAs, the radius of the zone of inhibition (RAD) is reverse proportional to MIC (Milici et al., 2007). Here, we found that none of the adaptors had an obvious change in RAD as compared to the parent (Figure 2B). *diskImageR* analysis of DDA pictures indicated the parent, the Chr1x3 adaptor, and the Chr5x1 adaptor had the same RAD value of 9.5 ± 0.7. When tested with another echinocandin drug micafungin (MCF), the parent and the adaptors had the same RAD value of 12.0 ± 0.0. Therefore, the CSP adaptors did not develop resistance to CSP or MCF (Figure 2B). This is consistent with the finding that none of the adaptors had mutations of *FKS* genes, since *FKS* mutations in clinical isolates of *Candida* spp. usually cause increased resistance to CSP (Balashov et al., 2006; Garcia-Effron et al., 2010; Imtiaz et al., 2012; Beyda et al., 2014; Marti-Carrizosa et al., 2015; Naicker et al., 2016; Szymankiewicz et al., 2021).

To confirm the development of CSP resistance and *FKS* mutations were rare events, we repeated this experiment by testing more adaptors. Approximately 1 million cells of #12108 were spread on YPD plates supplemented with 400 ng/ml of CSP. Randomly 192 were tested with DDAs and none of them had reduced RAD (data not shown).

In *C. albicans*, strains tolerant to azole drugs fluconazole (FLC) (Rosenberg et al., 2018) or ketoconazole (Xu et al., 2021) have increased FoG_20_ when tested by DDA with disks containing azoles, as indicated by colonies growing inside of the zone of inhibition. However, here we found none of the CSP adaptors had colonies growing inside of the zone of inhibition (Figure 2B). Therefore, the CSP adaptors do not have reduced RAD_20_ or increased FoG_20_.

Previously we found spot assay was a sensitive method to detect improved ability to grow in the presence of CSP in *C. albicans* (Yang et al., 2019). Here, we investigated whether spot assay could detect CSP tolerance in the adaptors. Spot assay indicated that the Chr5x3 adaptor (TJ60) grew better than the parent in the presence of CSP and MCF. Furthermore, spot assay indicated that all 29 Chr5x3 adaptors grew better than the parent in the presence of CSP and MCF (Supplementary Figure 1). However, the Chr1x3 adaptor (TJ74) did not grow better than the parent (Figure 2C). Thus, all CSP adaptors were aneuploid and had fitness cost in rich medium in the absence of stress; however, Chr5x3 enabled better fitness in the presence of CSP and MCF.

### Trisomy of chromosome 5 causes cross-tolerance to 5-flucytosine

Previously we found aneuploidy caused cross-tolerance to unrelated stresses in *C. albicans* (Yang et al., 2013, 2019, 2021b), *C. parapsilosis* (Yang et al., 2021c), and *Cryptococcus neoformans* (Yang et al., 2021a). Here, we investigated whether the aneuploid CSP adaptors caused cross-tolerance to other antifungal drugs. One Chr5x3 adaptor (TJ60) and one Chr1x3 adaptor (TJ74) were compared to the parent. DDAs with disks containing FLC or 5FC were performed.

**Figure 3:**
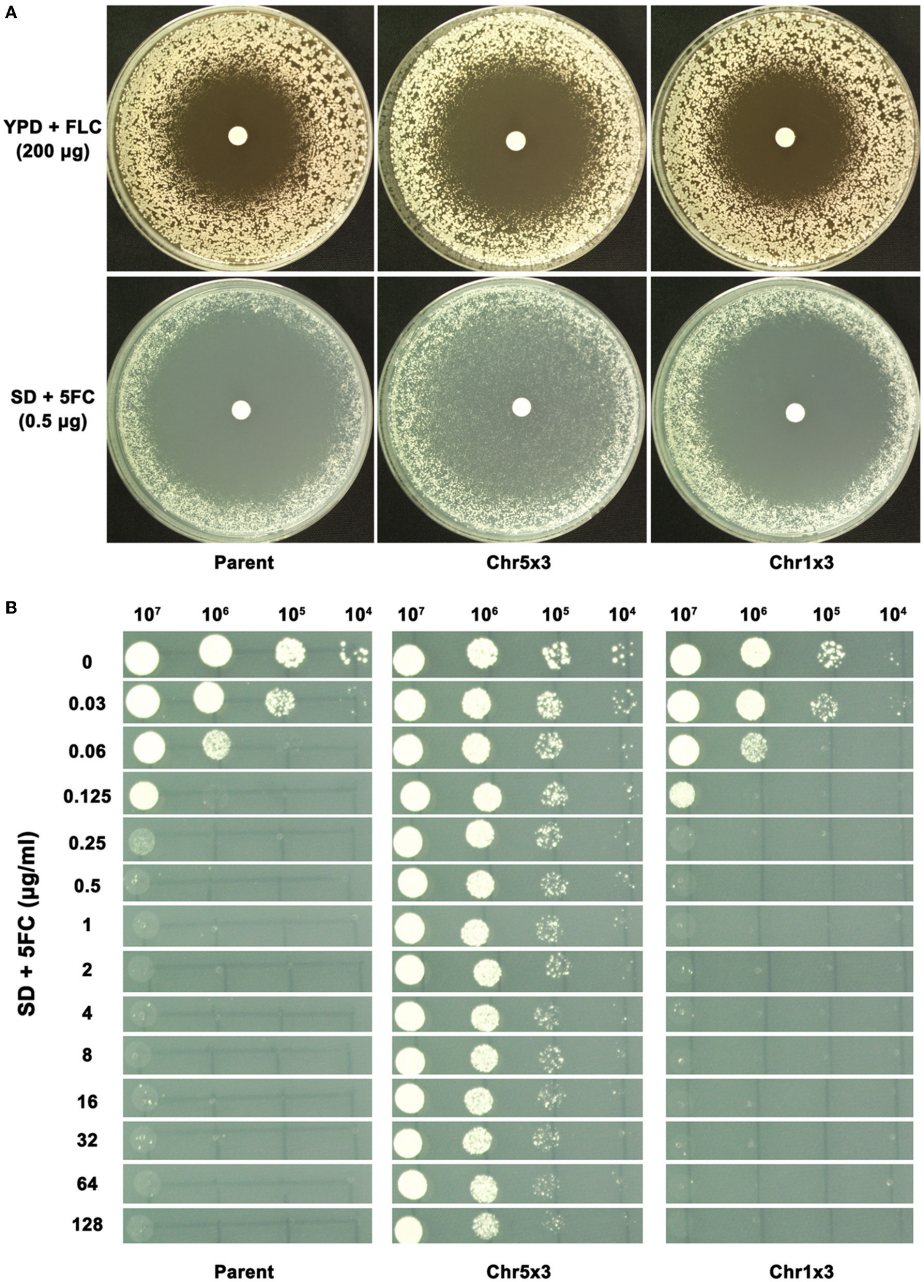
Chromosome 5 trisomy confers tolerance to 5-flucytosine. In **(A)**, disk diffusion assays were performed to compare aneuploids and the parent for tolerance to fluconazole (FLC) and 5-flucytosine (5FC). YPD and SD plates were used for testing FLC and 5FC, respectively. In **(B)**, spot assays were performed using a wide range of 5FC concentrations (0.03 μg/ml−128 μg/ml). A total of 3 μl of 10-fold serial dilutions were spotted on the plates. In **(A)** and **(B)**, the plates were incubated at 37°C for 72 h and then photographed.

When tested with FLC, the parent, Chr5x3 adaptor and Chr1x3 adaptor had a RAD_20_ of 17.0 ± 0.0, 23.5 ± 0.7, and 18.5 ± 0.7, respectively (Figure 3A). Therefore, Chr5x3 caused hypersensitivity to FLC, and Chr1x3 caused slightly increased sensitivity to FLC. When tested with 5FC, the parent, Chr5x3 adaptor, and Chr1x3 adaptor did not show an obvious change in RAD. However, inside the zone of inhibition, the Chr5x3 adaptor had lawn growth, while the Chr1x3 adaptor and the parent exhibited clear zones (Figure 3A). We investigated to what extent Chr5x3 can tolerate 5FC. Spot assay indicated that the growth of both parent and Chr1x3 was completely inhibited by 0.25 μg/ml of 5FC on the SD plate, but Chr5x3 could tolerate up to 128 μg/ml of 5FC (Figure 3B). Therefore, Chr5x3 caused hypersensitivity to FLC and tolerance to 5FC.

### Cross-tolerance to caspofungin and 5-flucytosine is controlled by chromosome 5 copy number

**Figure 4:**
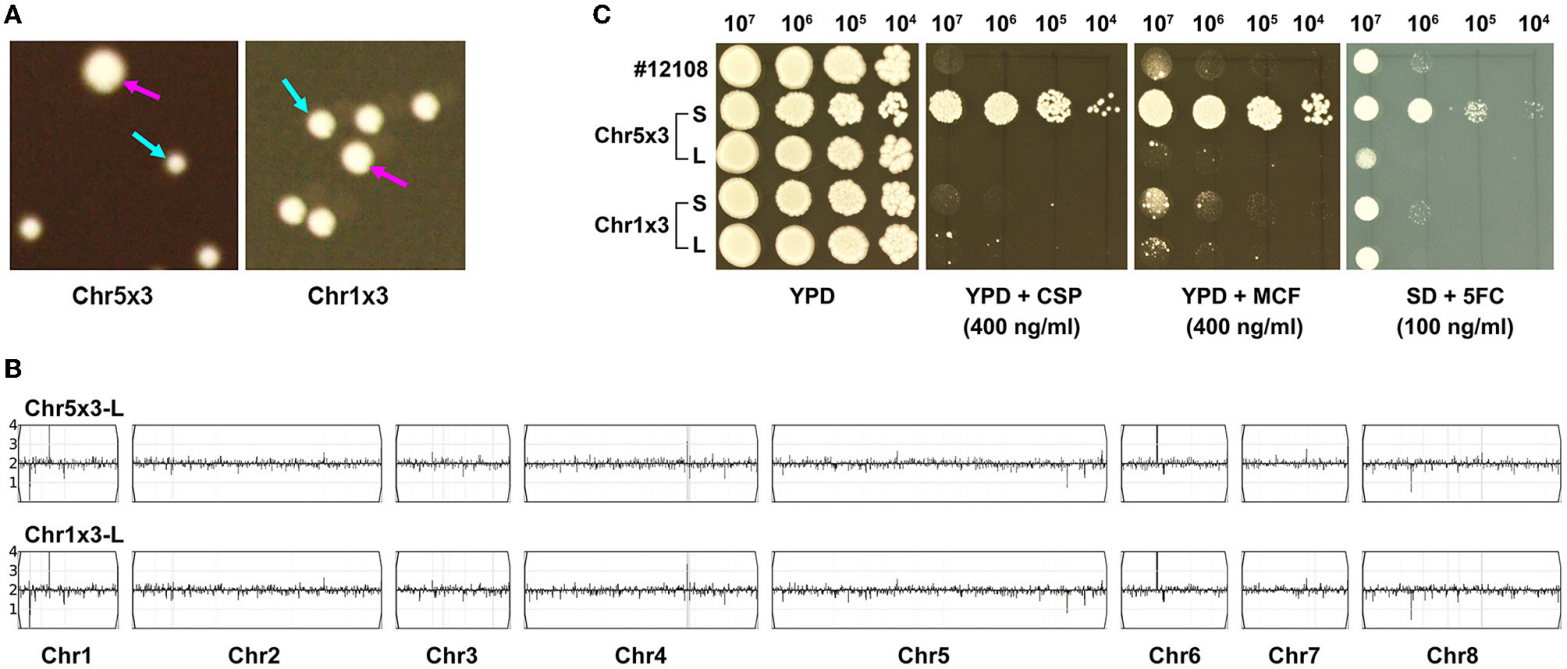
Genome and phenotype instability of aneuploids. Approximately 200 cells of aneuploid adaptors were spread on YPD plates. The plates were photographed after 72 h of incubation at 37°C. Cyan arrows indicate small (S) colonies and magenta arrows indicate large (L) colonies **(A)**. The L colonies were sequenced, and the karyotypes were visualized using Ymap **(B)**. For each adaptor, S and L colonies were compared for tolerance to caspofungin (CSP), micafungin (MCF), and 5-flucytosine (5FC) **(C)**. Spot assays were performed using YPD plates supplemented with CSP or MCF, and the SD plate supplemented with 5FC. The plates were incubated at 37°C for 72 h and then photographed.

We asked if aneuploids in *C. parapsilosis* were unstable. Approximately 200 cells of the Chr5x3 adaptor (TJ60) and Chr1x3 adaptor (TJ74) were spread on YPD plates. After incubation at 37°C for 48h, both adaptors exhibited colony instability: most colonies were small (indicated by cyan arrows in Figure 4A) and a few colonies were large (indicated by magenta arrows in Figure 4A). Whole-genome sequencing indicated that the large colonies were euploids (Figure 4B). Spot assay indicated that only the small colony of the Chr5x3 adaptor was tolerant to CSP, MCF, and 5FC. The large colony was not tolerant (Figure 4C). We investigated whether all 30 adaptors (TJ60–TJ89) were unstable. We found that all of them yielded small and large colonies on YPD plates (data not shown). Spot assay indicated that none of the large colonies was tolerant to CSP (Supplementary Figure 2). Therefore, in *C. parapsilosis*, aneuploids (Chr5x3 and Chr1x3) are unstable. Increased copy number of Chr5 causes tolerance to echinocandins and cross-tolerance to 5FC. Reversion of Chr5x3 to Chr5x2 is accompanied by the loss of tolerance and cross-tolerance to antifungal drugs.

### Aneuploidy causes the proportional change in transcriptomes

**Figure 5:**
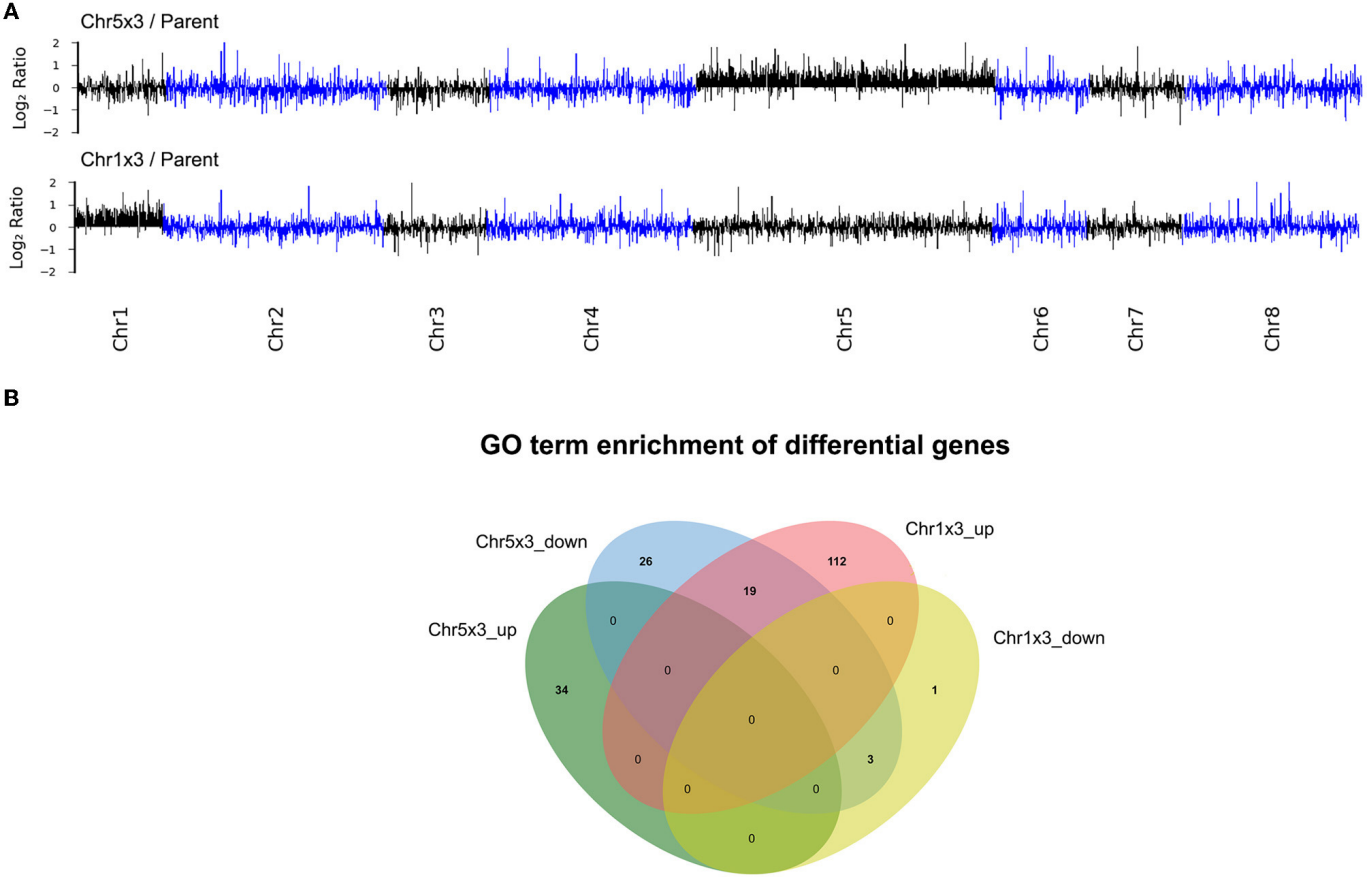
Transcriptional consequences of aneuploidy. Transcriptomes of Chr5x3 and Chr1x3 adaptors were compared to the diploid parent. Log_2_ ratios were plotted as a function of gene position on the chromosomes **(A)**. Differential genes were analyzed for gene ontology (GO) enrichments. Lists of up and downregulated genes were analyzed separately. Venn diagram was used to illustrate the difference and commonality of the GO enrichments in genes upregulated in Chr1x3 (Chr1x3_up) and Chr5x3 (Chr5x3_up), as well as genes downregulated in Chr1x3 (Chr1x3_down) and Chr5x3 (Chr5x3_down) as compared to parent **(B)**.

Since the CSP adaptors do not have *FKS* mutations, and *FKS* genes are not on the aneuploid chromosome, we investigated the mechanism of drug tolerance by performing RNA-seq and we compared the transcriptomes of CSP adaptors to the parent. Among the 1412 ORFs on Chr5, 744 were differentially expressed genes (*q* < 0.05) in the Chr5x3 adaptor as compared to the parent. Only 14 of the 744 ORFs were downregulated. All the remaining ORFs (98.1%) were upregulated. Among the 375 ORFs on Chr1, 253 were differentially expressed genes (*q* < 0.05) in the Chr1x3 adaptors as compared to the parent. All of them (100%) were upregulated. Therefore, both Chr1x3 and Chr5x1 caused proportional elevated transcription of the genes on the aneuploid chromosomes (Figure 5A).

Among genes upregulated in the Chr5x3 adaptor, processes associated with peroxisome, DNA repair, cell cycle, and catabolism were significantly enriched. In contrast, among genes upregulated in the Chr1x3 adaptor, processes associated with biosynthesis including organic substances, cellular nitrogen compounds, and macromolecules were significantly enriched (Supplementary Table 3). Several processes were commonly downregulated in Chr5x3 and Chr1x3 adaptors, including protein folding (GOID: 0006457), mitochondrion organization (GODI: 0007005), and protein import into mitochondrial intermembrane space (GOID: 0045041). Some GO terms were significantly enriched in Chr5x3 downregulated genes and enriched in Chr1x3 upregulated genes, including ribosome biogenesis (GOID: 0042254), ribosome localization (GOID: 0033750), ribosomal subunit export from the nucleus (GOID: 0000054), peptide biosynthetic process (GOID:0043043), and translation (GOID: 0006412) (Figure 5B).

We investigated whether genes associated with β-1,3-glucan synthesis and chitin synthesis and degradation were differentially regulated in the Chr5x3 adaptor TJ60. None of the *FKS* genes (*GSC1, GSL1*, and *GSL2*) was upregulated in the Chr5x3 adaptor. Among the genes encoding chitin synthase (*CHS1*/*CPAR2_800050, CHS2/CPAR2_701490, CHS3/CPAR2_801800, CHS4/CPAR2_807030, CHS5/CPAR2_210990, CHS7/CPAR2_212710*, and *CHS8/CPAR2_502940*), only one gene, *CHS7* was significantly upregulated in the Chr5x3 adaptor. *CHS7* is on Chr5. Among the genes encoding chitinases (*CHT2*/CPAR2_502140, *CHT3*/*CPAR2_200660*, and *CHT4*/*CPAR2_211950*), two genes, *CHT3* and *CHT4* are on Chr5 but their transcription was compensated to the disomic level (Supplementary Table 4). Therefore, we posit that Chr5x3 enables tolerance to CSP *via* increasing copy number and transcription of *CHS7* and reducing the transcription of *CHT3* and *CHT4*, thereby increasing chitin content in the cell wall.

We also investigated whether genes associated with 5FC tolerance were downregulated in the Chr5x3 adaptor. *FUR1* was significantly downregulated in the Chr5x3 adaptor. *FCA1* and *FCY2* did not exhibit obvious change (Supplementary Table 4). *FUR1* is on Chr7. We posit that Chr5x3 causes 5FC tolerance *via* indirectly downregulating the transcription of the *FUR1* gene on the euploid chromosome.

Compared to parent, several *ERG* genes were downregulated in Chr5x3 adaptor, but not in Chr1x3 adaptor, including *ERG3*/*CPAR2_105550, ERG5*/*CPAR2_703970, ERG8*/*CPAR2_400710*, and *ERG11*/*CPAR2_303740*. *ERG11* was upregulated in the Chr1x3 adaptor. *ERG1*/*CPAR2_210480, ERG4*/*CPAR2_502980*, and *ERG13*/*CPAR2_701400* were upregulated in the Chr1x3 adaptor, but not in the Chr5x3 adaptor. *ERG12/CPAR2_803530* was downregulated in both Chr1x3 and Chr5x3 adaptors. *UPC2*/*CPAR2_207280* encodes a transcription factor that positively regulates ergosterol biosynthetic genes. In Chr5x3 but not in the Chr1x3 adaptor, *UPC2* was upregulated. In addition, *CDR1/CPAR2_405290*, which encodes the drug efflux pump, was downregulated in the Chr5x3 adaptor, but not in the Chr1x3 adaptor, as compared to the parent (Supplementary Table 4). Taken together, we posit that Chr5x3 causes hypersensitivity to FLC *via* decreasing the expression of *ERG* genes and *CDR1*.

We visualized the genome sequencing data of the Chr5x3 adaptor and the parent in IGV, and we did not see mutations of genes *ERG3, ERG11*, or *CDR1*

The expression profile of genes in RNA-seq results was validated by reverse transcriptase PCR (RT-PCR). Six genes were tested, including *CHS7, CHT3, CHT4, FUR1, ERG11*, and *CDR1* (Supplementary Figure 4).

### Chr1x3 strain adapts to caspofungin mainly *via* amplification of Chr5

**Figure 6:**
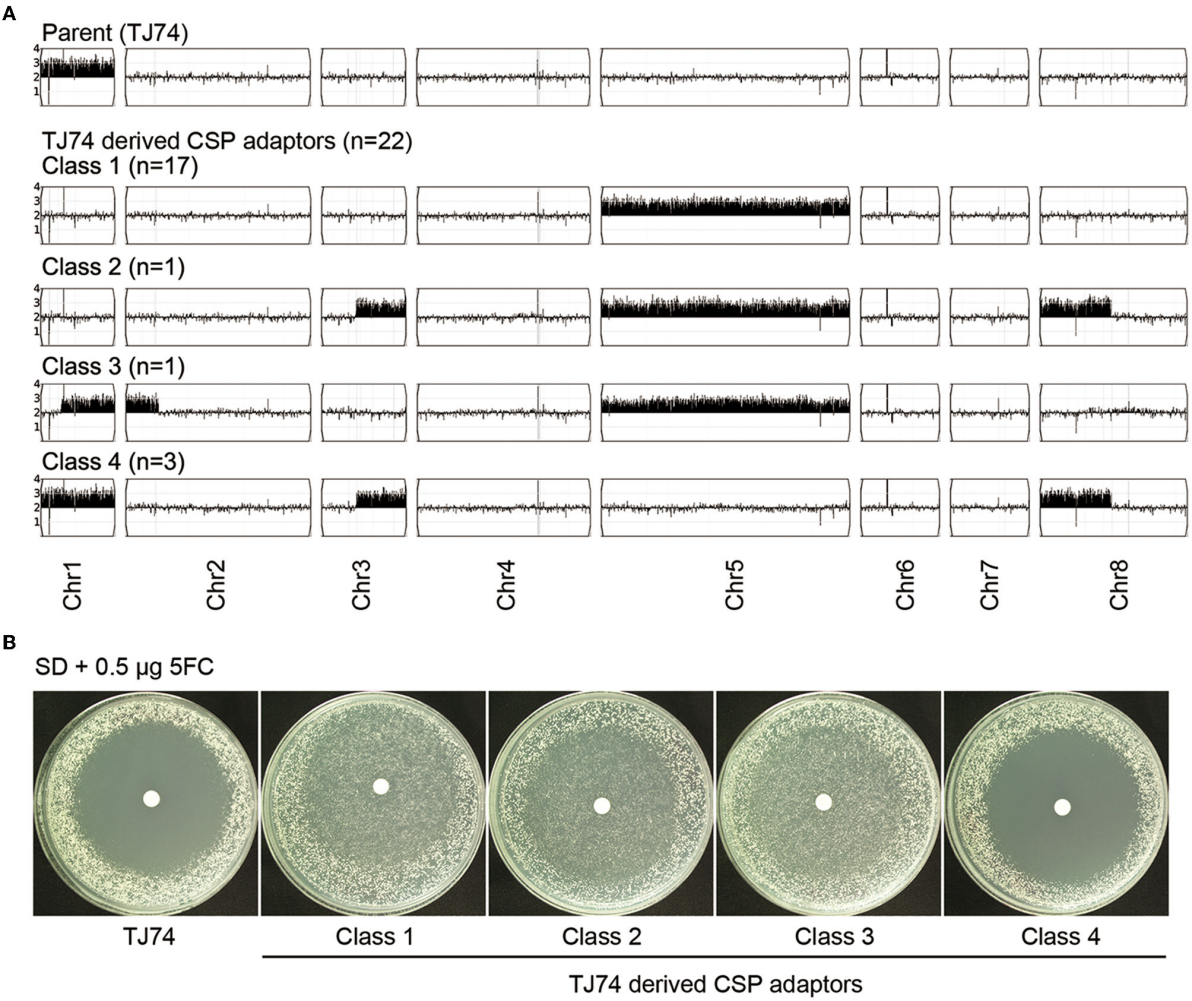
Adaptation of chromosome 1 trisomy strain to caspofungin. One chromosome 1 trisomy strain, TJ74, was spread on the YPD plate supplemented with 400 ng/ml caspofungin (CSP). Twenty-two out of 30 adaptors randomly chosen gained tolerance to CSP. All 22 tolerant adaptors were sequenced. Based on the pattern of aneuploidy, the adaptors were divided into four classes **(A)**. Each class was tested for tolerance to 5-flucytosine (5FC) by disk diffusion assays. Only adaptors with Chr5x3 were tolerant to 5FC **(B)**.

We investigated how a Chr1x3 strain would adapt to CSP. Approximately one million cells of adaptor TJ74 were spread on the YPD plate supplemented with 400 ng/ml of CSP. Randomly 30 adaptors (TJ2267–TJ2296) were chosen. Spot assay indicated that 22 of the 30 adaptors grew better than TJ74 and #12108 in the presence of CSP (Supplementary Figure 3). We sequenced all 22 tolerant adaptors. Based on the karyotypes, the tolerant adaptors were categorized into four classes: Class 1 adaptors (*n* = 17) had Chr5x3 alone. Class 2 adaptor (*n* = 1) had Chr5x3, segmental trisomy of Chr3 (SegChr3x3, from 0.43 Mb till the right telomere), and segmental trisomy of Chr8 (SegChr8x3, from left telomere till 0.87 Mb). Class 3 adaptor (*n* = 1) had Chr5x3, segmental trisomy of Chr1 (from 0.40 Mb till the right telomere), and segmental trisomy of Chr2 (SegChr2x3, from left telomere to 0.25 Mb). Class 4 adaptors (*n* = 3) had Chr1x3, SegChr3x3, and SegChr8x3 (Figure 6A). Of note, none of the 22 tolerant adaptors had *FKS* mutations. Although *GSC1* and *GSL1* are on Chr2, in the Class 3 adaptor, which had SegChr2x3, the amplified region of Chr2 did not encompass *GSC1* and *GSL1*. Therefore, the Chr1x3 strain adapted to CSP mainly *via* losing Chr1x3 and gaining Chr5x3 (19 out of 22) or maintaining Chr1x3 but gaining SegChr3x3 and SegChr8x3. Genetic mutation or elevated copy number of *FKS* genes did not happen.

We enquired whether Chr1x3-derived CSP adaptors were cross-tolerant to 5FC. DDAs on SD plates with paper disks containing 5FC indicated that all Class 1, Class 2, and Class 3 adaptors were tolerant to 5FC and Class 4 adaptors were not (Figure 6B). Therefore, adaptors with Chr5x3 were cross-tolerant to CSP and 5FC. Adaptors without Chr5x3 were only tolerant to CSP but not tolerant to 5FC.

## Discussion

In this study, we found that aneuploidy, Chr5x3 in particular, enabled rapid adaptation of both euploid and aneuploid *C. parapsilosis* strains to echinocandins and caused cross-tolerance to 5FC. Chr5x3 simultaneously upregulated the expression of genes on the aneuploid Chr5 and genes on euploid chromosomes. The large-scale effect of aneuploidy on whole-genome expression resulted in the pleiotropic effect on antifungal tolerance, including the development of cross-tolerance.

Of note, *FKS* mutations were not detected in the CSP adaptors, and *FKS* genes were not differentially expressed in the CSP adaptors as compared to the wild type. In *C. albicans*, increased expression of genes encoding chitin synthases or decreased expression of genes encoding chitinases causes tolerance to CSP (Walker et al., 2008; Suwunnakorn et al., 2016). Here we found that, among the genes encoding chitin synthases, *CHS7* is on Chr5 and was significantly upregulated in the Chr5x3 adaptor compared to the parent. The increased expression of *CHS7* might be due to increased copy number, since in yeast cells, aneuploidy usually caused the proportional change at the transcript level (Pavelka et al., 2010). Among the genes encoding chitinases, *CHT3* and *CHT4* are on Chr5, but the expressions of *CHT3* and *CHT4* in the Chr5x3 adaptor and in the parent were similar. Therefore, we posit that Chr5 confers CSP tolerance by upregulating the copy number and expression of *CHS7* to the trisomic level and buffering the expression of *CHT3* and *CHT4* to the disomic level. It remains unclear how Chr5x3 selectively buffers the expression of *CHT3* and *CHT4*, but not *CHS7*. Whether dosage compensation at the transcript level exists in yeast cells is still under debate (Hose et al., 2015; Torres et al., 2016).

Tolerance to 5FC in clinical isolates of *Candida* and *Cryptococcus* species is usually due to loss-of-function mutations of genes involved in the cellular uptake and intracellular metabolism of 5FC (Whelan, 1987; Hope et al., 2004; Papon et al., 2007; Billmyre et al., 2020; Chang et al., 2021). In *C. albicans*, the *FUR1* gene is on Chr5. Previously we found decreased copy number of *FUR1 via* Chr5x1 conferred tolerance to 5FC (Yang et al., 2013). In *C. parapsilosis, FUR1* is on Chr7. Here we found in *C. parapsilosis* that Chr5x3 downregulated the expression of *FUR1*. Thus, we posit on Chr5, there is a negative regulator of *FUR1* on Chr7 in the C. *parapsilosis* genome, and Chr5x3 confers 5FC tolerance *via* downregulating the expression of *FUR1*.

The Chr5x3 adaptor was more susceptible to FLC, indicating that aneuploidy *per se* does not cause tolerance to antifungal agents. Altered ergosterol synthesis and drug efflux cause FLC tolerance in *Candida* spp. (Berkow and Lockhart, 2017). Here we found that several *ERG* genes were downregulated in the Chr5x3 adaptor, including *ERG3, ERG5, ERG8, ERG11*, and *ERG12*. In addition, *CDR1*, which encodes the drug efflux pump, was also downregulated.

Taken together, Chr5x3 enables cross-tolerance to CSP and 5FC and hypersensitivity to FLC *via* simultaneously regulating genes associated with antifungal tolerance on aneuploid chromosomes and on the remaining euploid chromosomes.

In fungi, aneuploids are inherently unstable. In *C. albicans, C. auris*, and *Cryptococcus neoformans*, drug-tolerant aneuploids spontaneously revert to euploids in the absence of a drug, and tolerance to drugs is concomitantly lost (Sionov et al., 2010; Yang et al., 2019; Bing et al., 2020). Here we found that aneuploid *C. parapsilosis* adaptors were also unstable. Single-time growth on the YPD plate was sufficient to induce the reversion of Chr1x3 and Chr5x3 adaptors to euploids. Cross-tolerance to CSP and 5FC in the Chr5x3 adaptor was concomitantly lost after it reverted to euploid. Therefore, in *C. parapsilosis*, aneuploidy is a reversible strategy of adaptation to stresses including antifungal drugs.

## Conclusion

In summary, this study indicates that Chr5x3 is the major mechanism of rapid and reversible adaptation to CSP and cross-adaptation to 5FC in *C. parapsilosis*. The pleiotropic effect of aneuploidy on antifungal tolerance is *via* directly regulating genes on the aneuploidy chromosome and indirectly regulating genes on the euploid chromosomes. It will be interesting to investigate whether the formation of Chr5x3 happens in infected patients treated with CSP.

## Data availability statement

The datasets presented in this study can be found in online repositories. The names of the repository/repositories and accession number(s) can be found below: https://www.ebi.ac.uk/arrayexpress/, E-MTAB-12335; https://www.ebi.ac.uk/arrayexpress/, E-MTAB-12321; https://www.ebi.ac.uk/arrayexpress/, E-MTAB -12322.

## Author contributions

FY and Y-yJ analyzed the data. FY wrote the manuscript. L-lS and HL carried out the research. T-hY and Y-bC helped to develop the experimental idea and design. Y-yJ and Y-bC funded the experiments. All authors approved the submitted version.
