# Supplementary figures and images for: Aneuploidy enables cross-tolerance to unrelated antifungal drugs in Candida parapsilosis
Source: Front Microbiol. 2023 Apr 11;14:1137083. doi: 10.3389/fmicb.2023.1137083 (PMC10126355; doi:10.3389/fmicb.2023.1137083)

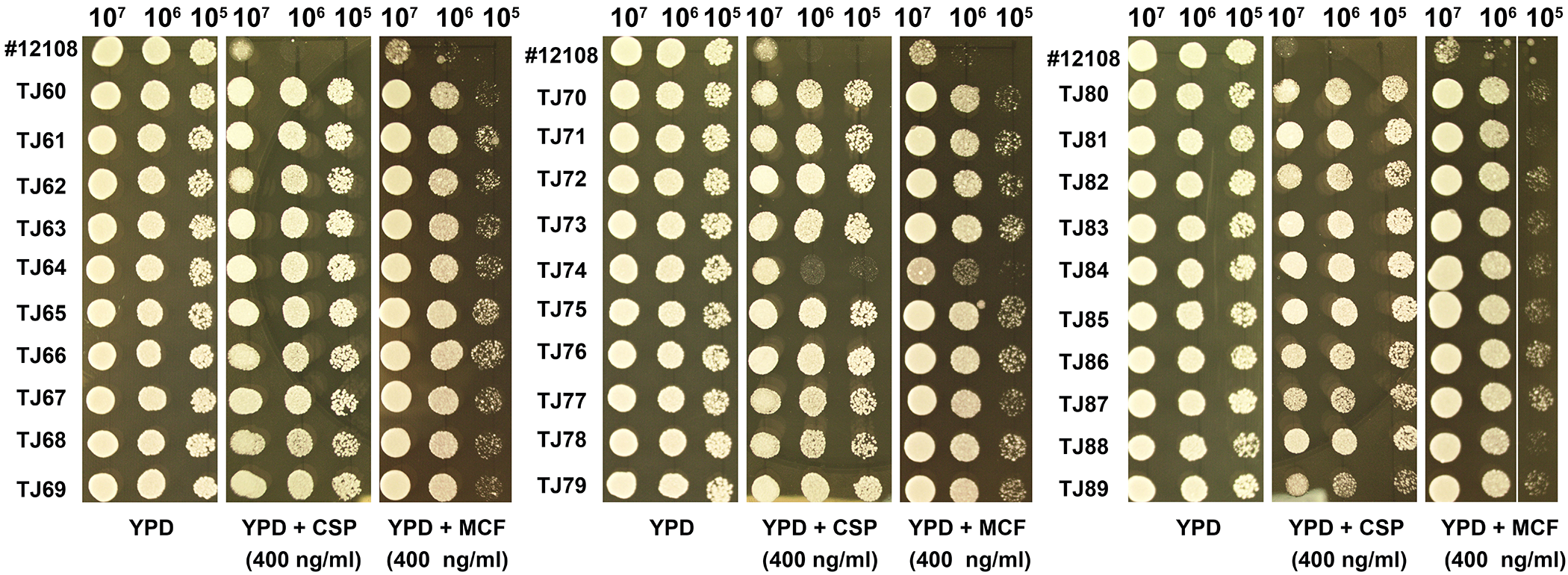

Supplement: Supplementary file 1 [file Image_1.TIF]

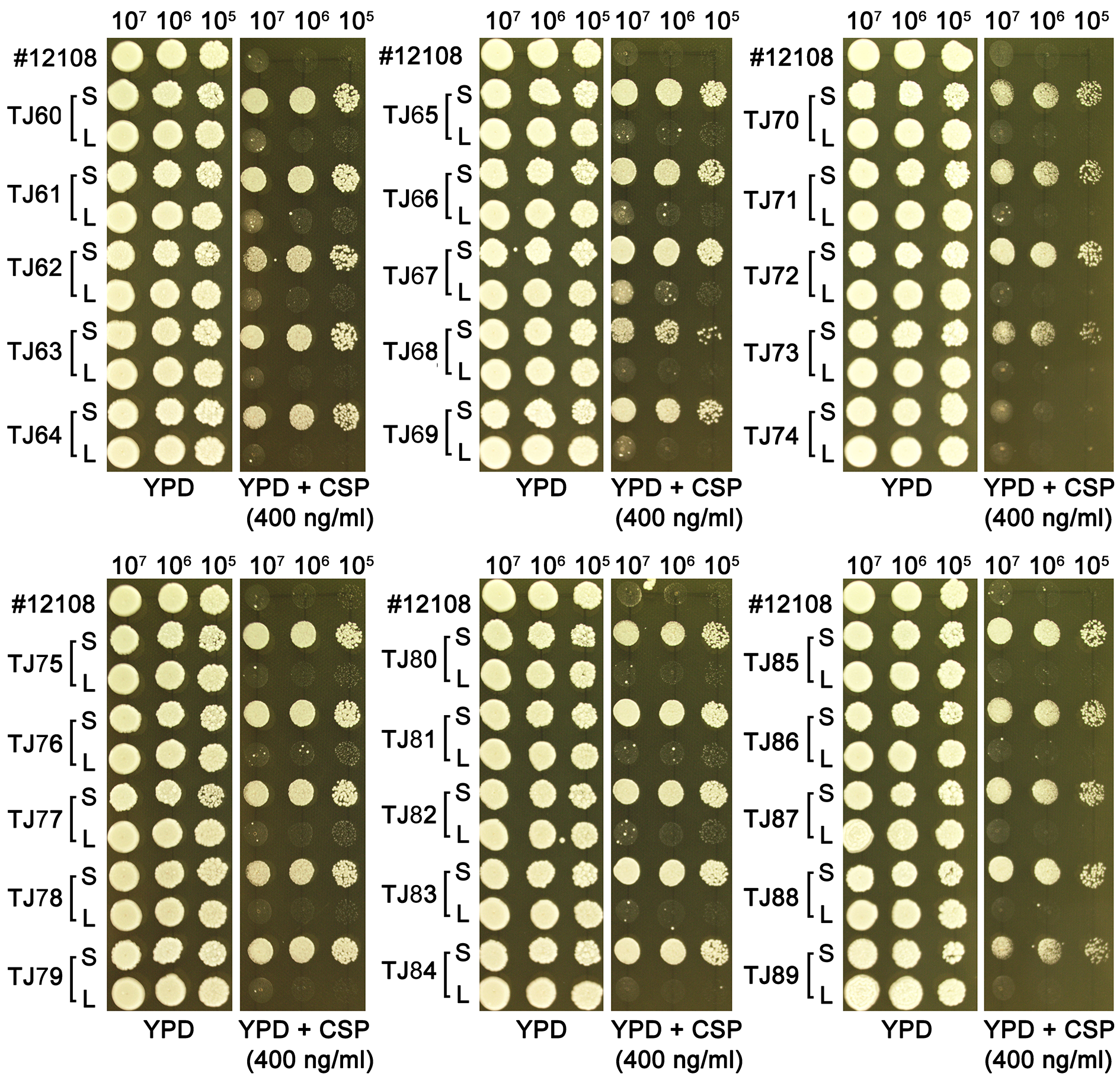

Supplement: Supplementary file 2 [file Image_2.TIF]

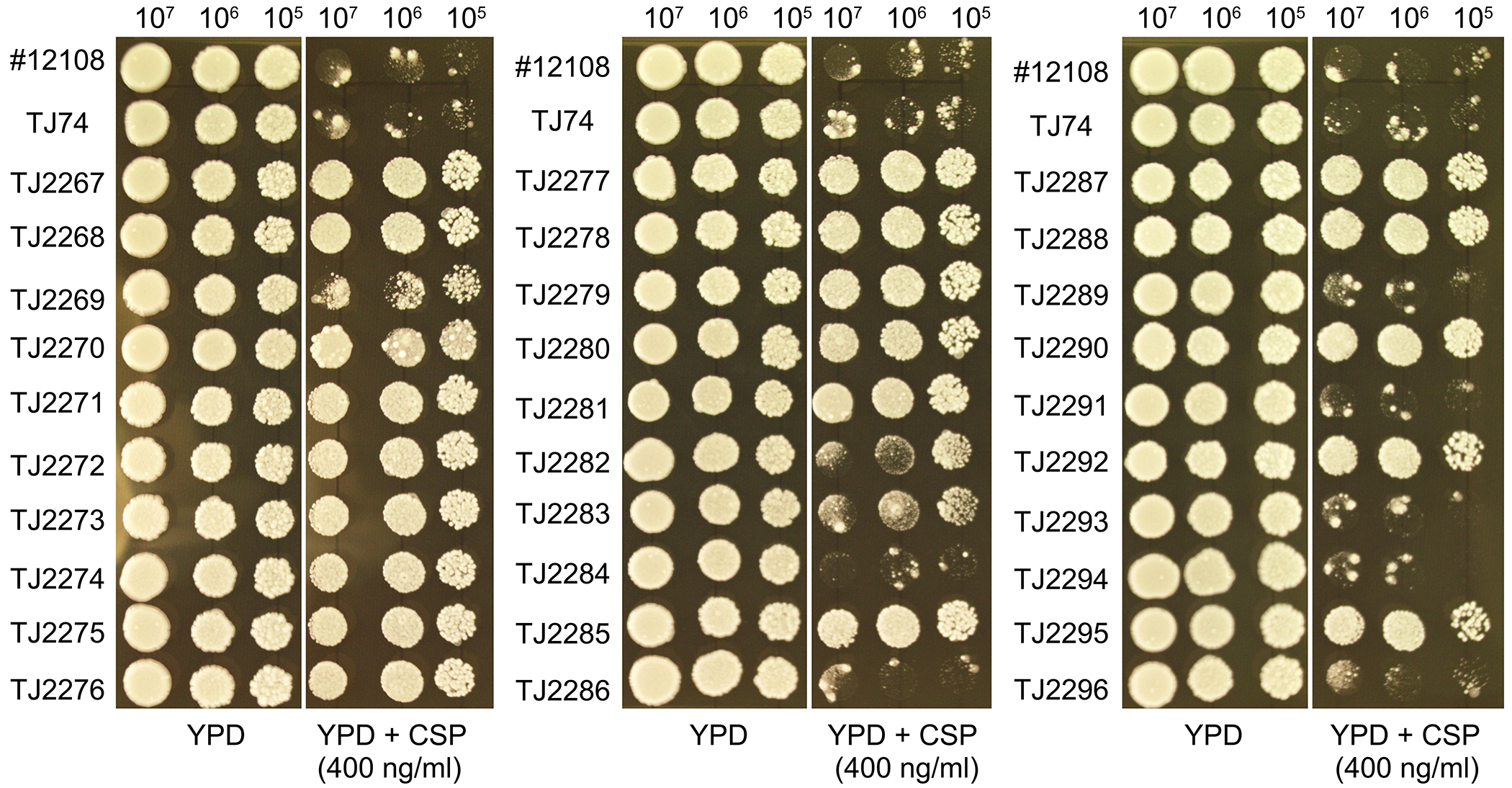

Supplement: Supplementary file 3 [file Image_3.TIF]

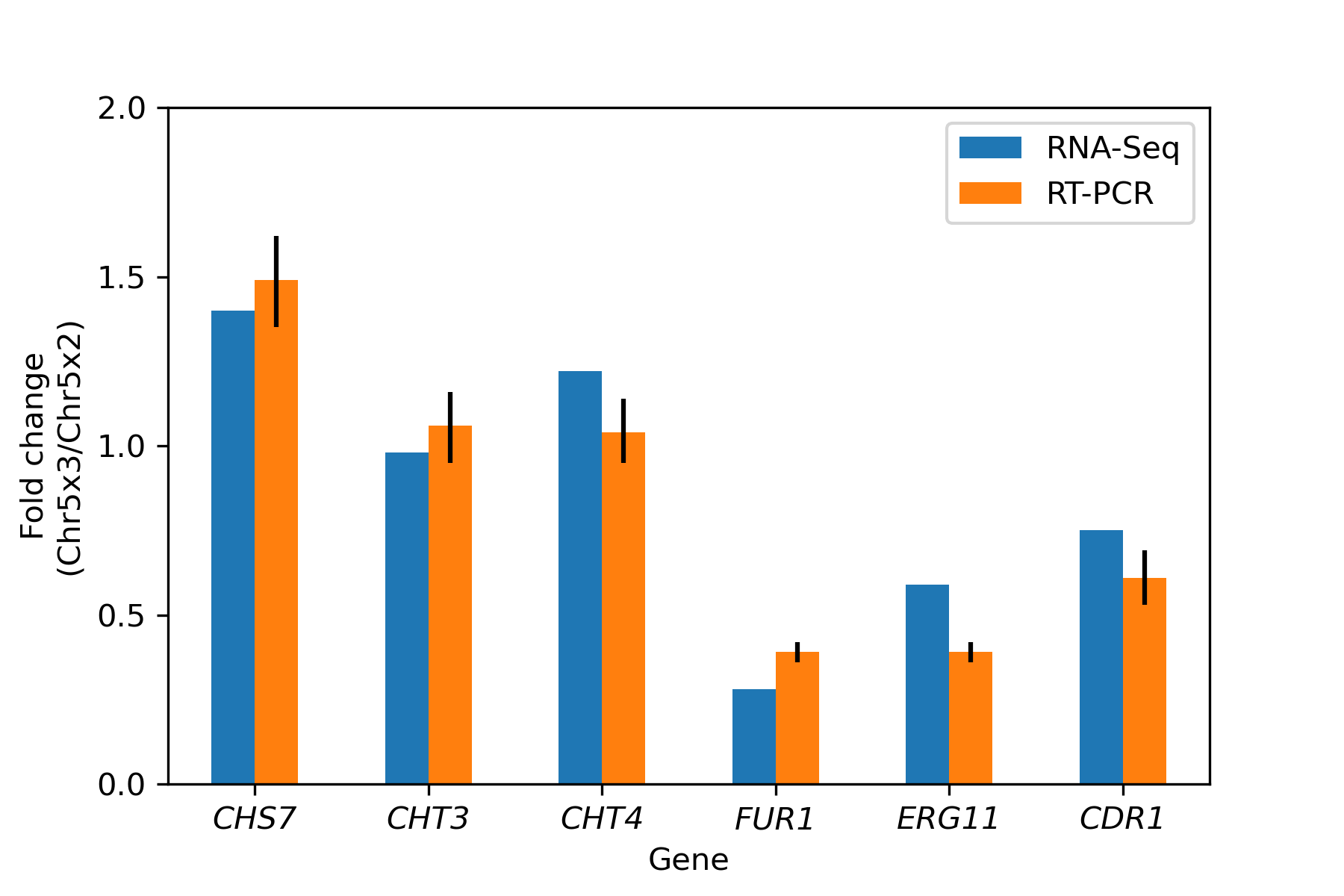

Supplement: Supplementary file 4 [file Image_4.TIFF]
