# Supplementary material for: Aneuploidy enables cross-tolerance to unrelated antifungal drugs in Candida parapsilosis
Source: Front Microbiol. 2023 Apr 11;14:1137083. doi: 10.3389/fmicb.2023.1137083 (PMC10126355; doi:10.3389/fmicb.2023.1137083)
Supplement: Supplementary file 5 [file Table_1.DOCX]

Table S1. Strains used in this study

| Name | Karyotype | Source |
| --- | --- | --- |
| #12584 | Wild type diploid | Clinical isolate |
| TJ60-TJ73 | Chr5x3 | CSP adaptors derived from #12584 |
| TJ74 | Chr1x3 | CSP adaptors derived from #12584 |
| TJ75-TJ89 | Chr5x3 | CSP adaptors derived from #12584 |
| TJ2267, TJ2268, TJ2271-TJ2279, TJ2285,TJ2287, TJ2288,TJ2290, TJ2292, TJ2295 | Chr5x3 | CSP adaptors derived from TJ74 |
| TJ2269, | SegChr3x3+Chr5x3+SegChr8x3 | CSP adaptors derived from TJ74 |
| TJ2280 | SegChr1x3+SegChr2x3+Chr5x3 | CSP adaptors derived from TJ74 |
| TJ2282, TJ2283, TJ2296 | Chr1x3+SegChr3x3+SegChr8x3 | CSP adaptors derived from TJ74 |
