# Supplementary material for: Aneuploidy enables cross-tolerance to unrelated antifungal drugs in Candida parapsilosis
Source: Front Microbiol. 2023 Apr 11;14:1137083. doi: 10.3389/fmicb.2023.1137083 (PMC10126355; doi:10.3389/fmicb.2023.1137083)
Supplement: Supplementary file 6 [file Table_2.DOCX]

Table S2. Primers used in this study

| Primer | Sequence (5’ 🡪 3’) |
| --- | --- |
| ACT1-Fwd | CGAACGTGGTTACGGTTTCT |
| ACT1-Rev | TGACCATCTGGCAATTCGTA |
| CHS7-Fwd | CATCCTATTTCCAGCGTGGT |
| CHS7-Rev | ACGCCACAATCAACAATCAA |
| CHT3-Fwd | ATGAAACGCTTGGTTGGAAG |
| CHT3-Rev | GCTGGTGGAGCTTGAAGAAC |
| CHT4-Fwd | AACCGTTCATGGAAGTTTGC |
| CHT4-Rev | TCGAATCCGTACTCCTCCAC |
| FUR1-Fwd | CAATGATGGCAGTGGAAGTG |
| FUR1-Rev | CAAAATCTCCCAAACCAGGA |
| ERG11-Fwd | TGTTGCATTTGGCTGAGAAG |
| ERG11-Rev | TCTGAGGGTTTCCTTGATGG |
| CDR1-Fwd | TTGGCGTTTTTGTGTTGGTA |
| CDR1-Rev | GGCAAGATTGGCAGCATTAT |
