# Supplementary material for: Aneuploidy enables cross-tolerance to unrelated antifungal drugs in Candida parapsilosis
Source: Front Microbiol. 2023 Apr 11;14:1137083. doi: 10.3389/fmicb.2023.1137083 (PMC10126355; doi:10.3389/fmicb.2023.1137083)
Supplement: Supplementary file 8 [file Table_4.DOCX]

Table S4. Relative expression of some genes

| Systematic name | Gene | Ratio | |
| --- | --- | --- | --- |
|  |  | Chr5x3/Parent | Chr1x3/Parent |
| **β-1,3-glucan synthase genes** | | | |
| *CPAR2_106400* | *GSC1* | 0.98 | 0.93 |
| *CPAR2_109680* | *GSL1* | 1.17 | 1.08 |
| *CPAR2_804030* | *GSL2* | 1.11 | 0.90 |
| **Chitin synthase genes** | | | |
| *CPAR2_805640* | *CHS1* | 1.00 | 0.96 |
| *CPAR2_701490* | *CHS2* | 1.28 | 1.00 |
| *CPAR2_801800* | *CHS3* | 1.09 | 1.09 |
| *CPAR2_807030* | *CHS4* | 0.85 | 1.04 |
| *CPAR2_210990* | *CHS5* | 1.16 | 0.94 |
| *CPAR2_303060* | *CHS6* | 0.94 | 1.02 |
| *CPAR2_212710* | *CHS7* | **1.40*** | 0.93 |
| *CPAR2_502940* | *CHS8* | 0.95 | 0.84 |
| **Chitinase genes** | | | |
| *CPAR2_800050* | *CHT1* | 0.68 | 0.85 |
| *CPAR2_502140* | *CHT2* | 0.85 | 1.54 |
| *CPAR2_200660* | *CHT3* | 0.98 | 0.86 |
| *CPAR2_211950* | *CHT4* | 1.22 | 0.77 |
| **Genes associated with 5FC tolerance** | | | |
| *CPAR2_502030* | *FUR1* | **0.28*** | 0.85 |
| *CPAR2_602820* | *FCA1* | 0.70 | **1.60*** |
| *CPAR2_806580* | *FCY2* | 0.94 | 1.16 |
| **Genes associated with FLC tolerance** | | | |
| *CPAR2_210480* | *ERG1* | 1.18 | **1.34*** |
| *CPAR2_105550* | *ERG3* | **0.54*** | 0.90 |
| *CPAR2_502980* | *ERG4* | 1.08 | **1.32*** |
| *CPAR2_703970* | *ERG5* | **0.76*** | 0.94 |
| *CPAR2_400710* | *ERG8* | **0.76*** | 0.89 |
| *CPAR2_303740* | *ERG11* | **0.59*** | **1.22*** |
| *CPAR2_803530* | *ERG12* | **0.65*** | **0.72*** |
| *CPAR2_701400* | *ERG13* | 0.83 | **1.35*** |
| *CPAR2_207280* | *UPC2* | **1.29*** | 0.90 |
| *CPAR2_405290* | *CDR1* | **0.75*** | 1.20 |

^*^q<0.05
